# Efficacy of a Commercial PRRSV Vaccine on NADC34-Like PRRSV Challenge

**DOI:** 10.1155/2023/4509261

**Published:** 2023-06-29

**Authors:** Lili Yuan, Zhenbang Zhu, Juan Fan, Qun Li, Panrao Liu, Xiangdong Li

**Affiliations:** ^1^Jiangsu Co-innovation Center for Prevention and Control of Important Animal Infectious Diseases and Zoonoses, College of Veterinary Medicine, Yangzhou University, Yangzhou, China; ^2^Joint International Research Laboratory of Agriculture and Agri-Product Safety, The Ministry of Education of China, Yangzhou University, Yangzhou, China; ^3^Yangzhou Uni-Bio Pharmaceutical Co., Ltd., Yangzhou, China

## Abstract

NADC34-like porcine reproductive and respiratory syndrome virus (PRRSV) has become endemic in some provinces of China and caused huge economic losses to local pig industry. The increased reports of NADC34-like PRRSV outbreaks in vaccinated pig herds suggest the limited protection of immunization with commercial vaccines. In this study, we evaluated a commercial PRRSV vaccine that has been widely used in China against the challenge of JS2021NADC34 PRRSV, a highly pathogenic Chinese NADC34-like strain isolated in 2021. The vaccinated pigs developed PRRSV-specific antibody responses, as shown by IDEXX ELISA results. After JS2021NADC34 PRRSV challenge, the vaccinated pigs had low level of viremia but suffered pathological lesions in lungs and lymphoid tissues. The viral antigens were also detected in the above tissues of the vaccinated pigs by immunohistochemistry staining. One out of five pigs in vaccinated group died at 13 days postchallenge. The above results suggested that the commercial PRRSV vaccine could not provide complete protection to the NADC34-like PRRSV infection.

## 1. Introduction

Porcine reproductive and respiratory syndrome virus (PRRSV) is the etiological agent of porcine reproductive and respiratory syndrome (PRRS), an important pig disease that led to huge economic loss to pig industry worldwide. Belonging to the order *Nidovirales*, family *Arteriviridae*, PRRSVs are classified into two genotypes, namely PRRSV-1 (European type or genotype I) and PRRSV-2 (North American type or genotype II). Both PRRSV-1 and PRRSV-2 are currently circulating in China with PRRSV-2 as the dominant genotype. PRRSV-2 could be further divided into nine lineages according to phylogenetic analysis of viral ORF5 sequences [1]. Among them, sublineage 1.8 (NADC30-like PRRSV) is the dominant PRRSV-2 strain in China with cocirculating of other PRRSVs belonging to lineage 8 (classical and highly pathogenic PRRSVs) [2].

Most recently, NADC34-like PRRSV, which belongs to sublineage 1.5, has drawn great attention since its frequent outbreaks in vaccinated pig herds in China [3]. However, the efficacy of commercial PRRSV vaccines on NADC34-like PRRSV was not experimentally evaluated before. Therefore, in this study, we immunized pigs with a commercial PRRSV vaccine and evaluated the vaccine efficacy to the challenge of JS2021NADC34 PRRSV, a highly pathogenic Chinese NADC34-like strain that was isolated in Jiangsu province in 2021 [4].

## 2. Materials and Methods

### 2.1. Virus and Cells

JS2021NADC34 PRRSV was propagated in porcine alveolar macrophages (PAMs), as previously described [5]. PAMs were obtained from 3-week-old specific pathogen-free pigs and cultured in RPMI 1640 medium (Gibco BRL Co., Ltd., USA) supplemented with 10% fetal bovine serum at 37°C in 5% CO_2_.

### 2.2. Animal Experiment

Ten 2-month-old large White-Duroc crossbred PRRSV-free pigs were randomly divided into two groups with five pigs in each group. The pigs in one group (five pigs) received immunization with a commercial live attenuated PRRSV vaccine (provided by Yangzhou Uni-Bio Pharmaceutical Co., Ltd.) as the manufacturer recommended. The pigs in another group received the vaccine diluent as placebos. Twenty-one days postvaccination (dpv), pigs in both groups were challenged with JS2021NADC34 PRRSV at 3 × 10^5^ median tissue culture infective dose (TCID_50_)/pig via intranasal (0.5 mL/nasal) and intramuscular (2 mL) routes simultaneously, as previously described [5]. Pigs were monitored daily for rectal temperatures and clinical signs. The pigs were humanly euthanized by pentobarbital sodium (intravenous injection, 150 mg/kg) when they had moribund conditions or at the end of the study which was terminated at 14 days postchallenge (dpc). The body weight gain of pigs was calculated, as previously described [5]. Animal experimental protocol was approved by the Animal Welfare and Ethic Committee of Yangzhou University with the reference number 202204002 and conventional animal welfare regulations and standards were taken into account.

### 2.3. Histopathology and Immunohistochemistry Staining

Lung, lymph node, and tonsil samples were collected at necropsy which were subjected to histopathology and immunohistochemistry staining, as previously described [5]. The staining was operated automatically by Leica fully automatic dyeing machine. The anti-PRRSV N (4A5) antibody (MEDIAN, Republic of Korea) was applied for immunohistochemistry staining.

### 2.4. Viremia and Serological Test

Blood samples of pigs were collected at designated days for detection of viremia and PRRSV-specific antibody. Total RNA was extracted from serum samples by using a RNeasy Mini kit (Qiagen, Germany) according to the manufacturer's instructions. The qPCR was performed, as previously described [5]. Serum samples were collected at designated days for viremia and IDEXX ELISA tests, as previously described [5].

## 3. Results and Discussion

**Figure 1:**
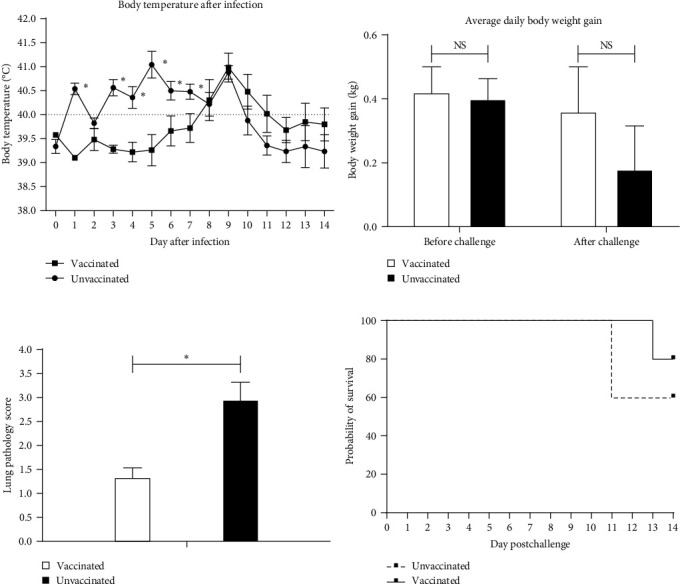
Body temperature (a), daily body weight gain (b), lung pathological scores (c), and survival rates (d) of pigs in two groups. The fever was set as above 40.0°C. The body weights of pigs were measured at 21 dpi and 14 dpc or on the day of pig was humanely euthanized. Gross lung lesion presented in all lung lobes at necropsy were scored using a 4-point scale. Raw data for body temperatures, body weight gains, and gross lung lesion scores were calculated and averaged.  ^*∗*^Indicated statistically significant difference (*p* < 0.05) and NS indicated not significant.

The vaccinated pigs stayed healthy and no clinical symptoms were observed before viral challenge. At 21 days postimmunization (dpi), all pigs were challenged with JS2021NADC34 PRRSV. The unvaccinated pigs developed consistent fever (body temperature ≥ 40.0°C) starting from 1 dpc (Figure 1(a)). By contrast, the vaccinated pigs developed fever starting at 6 dpc and all pigs had high fever (body temperature ≥ 40.5°C) at 9 dpc before body temperatures dropped back to normal at the end of the study. The daily body weight gain of pigs was calculated before and after viral challenge. As shown in Figure 1(b), there was no difference in daily body weight gain of pigs between vaccinated and unvaccinated groups. After viral challenge, the unvaccinated pigs had reduced daily body weight gain as compared with the vaccinated pigs but the difference was not significant. Besides fever, some other PRRSV-specific clinical symptoms including dehydration, respiratory distress, and shivering were also observed on pigs in both vaccinated and unvaccinated groups. There was no significant difference in the clinical scores between two groups (data not shown). Two out of five unvaccinated pigs were euthanized due to their moribund conditions at 11 dpc, and one out of five vaccinated pigs died at 13 dpc (Figure 1(d)). The rest of pigs were euthanized at 14 dpi.

At necropsy, gross pathological examination and scoring were performed, as previously described [5]. Pulmonary consolidations and necrosis in the lung were observed, and the gross pathological scores of lungs in unvaccinated groups were significantly higher than the ones in vaccinated groups (Figure 1(c)). Hemorrhage and necrosis were also observed in the tonsil and lymph nodes in both vaccinated group and unvaccinated group but the difference was not significant (data not shown).

**Figure 2:**
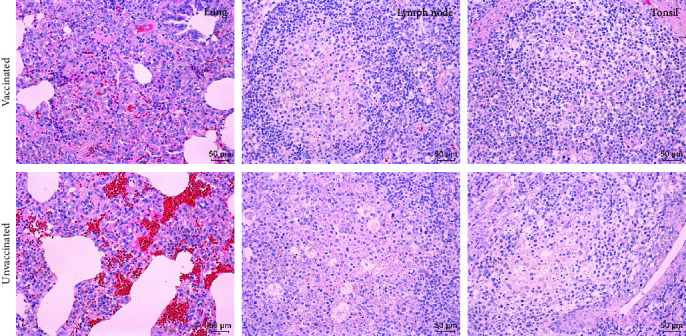
Histopathological examinations of pig lung, lymph node, and tonsil samples in two groups. (Left) Interstitial pneumonia with infiltration of mononuclear cells in the lungs. (Middle) Vascular dilatation and lymphatic tissue necrosis with lymphocyte depletion and infiltration of neutrophils in the lymph nodes. (Right) Tonsillar lymphoid tissue necrosis and formation of large necrotic foci in the tonsils. Original magnification, ×200.

**Figure 3:**
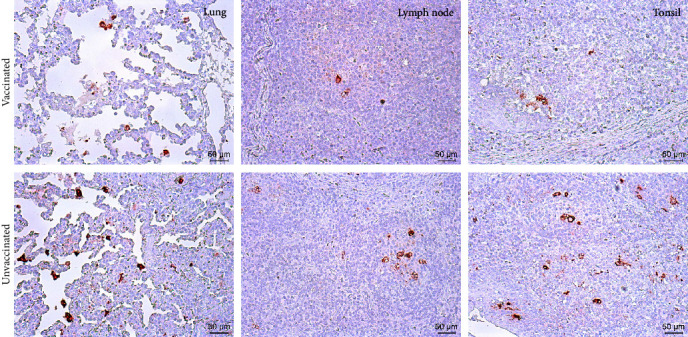
IHC staining of pig lung, lymph node, and tonsil samples in two groups. PRRSV antigens were recognized by a PRRSV-specific antibody and visualized in brown dots. Original magnification, ×200.

Histopathologically, pigs in both groups had interstitial pneumonia associated with hemorrhage, which were characterized by thickening of alveolar septa and infiltration of mononuclear cells (Figure 2). Besides histopathological changes of lungs, lymphocyte depletion, acute hemorrhage, and infiltration of neutrophils were also observed in lymph nodes and tonsils of pigs in both groups. By using a specific monoclonal antibody, PRRSV antigens were visualized in the above tissues in immunohistochemistry (IHC) staining (Figure 3). However, the numbers of positive cells in vaccinated pigs were reduced than the unvaccinated ones, and the intensity of positive staining was also less strong in vaccinated pigs.

**Figure 4:**
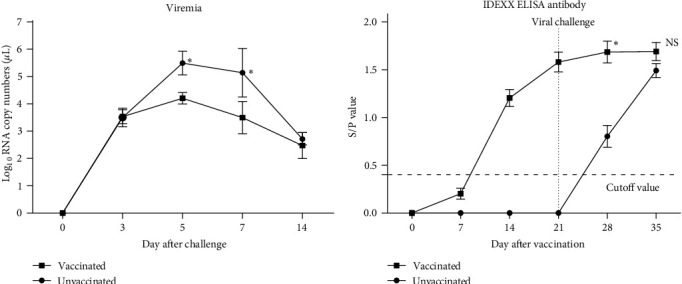
Viremia (a) and IDEXX PRRSV-specific antibodies (b) of pigs in two groups. PRRSV viral RNA in serum was determined by qPCR. The threshold of seroconversion for IDEXX ELISA was set at a sample-to-positive (S/P) ratio of 0.4 according to the manufacturer's instructions. ^ ^*∗*^^Indicated statistically significant difference (*p* < 0.05).

Pig serum samples were collected at designated days for viremia and IDEXX ELISA tests. As shown in Figure 4(a), the vaccinated pigs had significant lower viremia at 5 and 7 dpc but the difference was not significant at 14 dpc. The vaccinated pigs developed high IDEXX ELISA antibody titers with average S/P value above 1.5 at 21 dpi (Figure 4(b)). After viral challenge, the average S/P values of vaccinated pigs kept increasing. By contrast, the unvaccinated pigs developed PRRSV-specific antibodies quickly and reached similar S/P values as the vaccinated pigs did. Virus neutralizing (VN) antibodies of each pig to JS2021NADC34 PRRSV were measured at 21 dpv and 14 dpc, and no VN antibodies were detectable (VN titer ≤ 1 : 2) in all serum samples (data not shown).

The first report about NADC34-like PRRSV in China was in 2018 [3]. Since then, the outbreaks of this PRRSV strain were reported in at least nine provinces in China [2, 6, 7]. In these cases, NADC34-like PRRSV was reported to be responsible for the dramatic abortion storms in sows and high mortality rates in piglets, which indicated the inability of commercial vaccines to provide protection. Genetically, full-length genome sequence alignments revealed that NADC34-like PRRSV shared less than 85% sequence identity with VR-2332 (sublineage 5.1), CH-1a (sublineage 8.1), and JXA1 (sublineage 8.3) PRRSVs, the main vaccine strains used in China, which indicated the less cross-protection of commercial vaccines to NADC34-like PRRSV. Therefore, it is necessary to experimentally evaluate the efficacy of commercial PRRSV vaccines to NADC34-like PRRSV infection.

However, pathogenicity of different NADC34-like PRRSV isolates varies a lot due to the origin of viruses, recombination events, and patterns with other viral strains. JS2021NADC34 PRRSV, a Chinese NADC34-like PRRSV used in this study, was previously proved to be highly pathogenic to 2-month-old pigs by experimental infection [5]. After viral challenge, both unvaccinated and vaccinated pigs developed typical PRRSV clinical symptoms, and one vaccinated pig died at 13 dpc. Consistently, the results of viremia, histopathology, and IHC also proved the vaccination could not generate sterile immunity on pigs. Collectively, the above results indicated the commercial PRRSV vaccine could not provide complete protection to JS2021NADC34 PRRSV challenge.

One limitation of this study was that we did not test other commercial live attenuated PRRSV vaccines available in China due to the limited numbers of experimental pigs. However, the low genomic similarities of these vaccine strains with NADC34-like PRRSVs and frequent outbreaks in vaccinated pig herds make the efficacy of commercial PRRSV vaccines questionable.

## Data Availability

The data that support the findings of this study are available from the corresponding author upon reasonable request.
